# Micronutrient Status and Selected Physiological Parameters of Roots in Nickel-Exposed *Sinapis alba* L. Affected by Different Sulphur Levels

**DOI:** 10.3390/plants8110440

**Published:** 2019-10-23

**Authors:** Renata Matraszek-Gawron, Barbara Hawrylak-Nowak

**Affiliations:** Department of Botany and Plant Physiology, Faculty of Environmental Biology, University of Life Sciences in Lublin, Akademicka 15, 20-950 Lublin, Poland; renata.matraszek@up.lublin.pl

**Keywords:** metal toxicity, sulphur nutrition, stress mitigation, cation exchange capacity, glutathione

## Abstract

An efficient method of improving the micronutrient status of Ni-treated white mustard (*Sinapis alba* L.) using intensive S-SO_4_ nutrition was developed. Twelve variants of Hoagland’s nutrient solution differing in the concentration of S-SO_4_ (standard: 2 mM S, and elevated level: 6 or 9 mM S) and Ni (0, 0.0004, 0.04, or 0.08 mM Ni) were tested. The beneficial effect of intensive S nutrition on Ni-stressed plants was manifested by a significant rise in the content of Fe, Mn, and Zn, especially in the shoots. An increase was also found in the shoot B, Cu, and Mo content, whilst there were no changes in their root concentrations. Simultaneously, the shoot Cl concentrations dropped. The elevated level of S in the nutrient solution in general enhanced the translocation of Fe, Cu, Mo, and B in Ni-exposed plants. The beneficial effect of intensive S nutrition on the growth and micronutrient balance of Ni-exposed plants can be at least partially related to the positive changes in root surface properties, especially in cation exchange capacity (CEC). Meanwhile both reduced glutathione (GSH) and phytochelatins (PCs) probably do not significantly contribute to Ni resistance of white mustard under intensive S nutrition.

## 1. Introduction

Nickel, like other metallic micronutrients in plants, is a functional constituent of the enzyme systems and its role is primarily associated with the valence change. This element, at relatively low concentrations (0.001–0.01 mg kg^−1^ dry weight; DW) is needed for the proper N and C metabolism [1,2,3] as well as for producing high-vigor viable seeds and their germination [4,5]. The most common visual symptoms of Ni deficiency are growth reduction, senescence acceleration, and leaf deformation accompanied by chlorotic and necrotic lesions as a result of Fe deficiency induced in Ni-deficient plants [6,7]. However, not Ni deficiency, but its excess and strong phytotoxic effects are the serious global problem. Nickel may easily move in the environment. Of particular concern is the increasing area of Ni-contaminated agricultural soils together with rapidly rising Ni concentrations deposited in agricultural soils by airborne Ni particles. Moreover, the low soil pH as a result of reduced soil liming as well as acid rains may cause mobilization and enhance the solubility of Ni compounds [8,9]. It has been established that the Ni content in farm soils varies in a wide range from 3 to 1000 mg kg^−1^ DW. Most agricultural soils contain 25 mg kg^−1^; however, Ni content is very often raised, up to 26,000 mg kg^−1^ or even substantially higher, due to anthropogenic activities such as mining, smelting, burning of fossil fuels (coal and oil), use of industrial and municipal wastes (sewage sludge), as well as applications of pesticides and Ni-containing fertilizers, especially phosphates [8,9,10,11,12]. A strongly phytotoxic effect of high Ni concentrations manifests itself as growth and development inhibition including retarded germination, yield and quality reduction, as well as disturbances in photosynthesis, respiration, water relations, and sugar transport, which cause various ultrastructural modifications. Visual symptoms induced by the Ni excess include various types of leaf chlorosis, necrosis, and wilting [6,13,14,15]. These chlorotic and necrotic lesions are a result of altered essential nutrient uptake and translocation. Interference with nutrient homeostasis and thus improper nutrient, especially micronutrient, status within plants is mentioned as an important mechanism of Ni phytotoxicity [16,17]. The modifications of the mineral status of Ni-stressed plants within species and even cultivars are unpredictable and contradictory. Besides, it is very difficult to study the biological role and mechanisms of Ni toxicity due to the dual character and complicated electronic chemistry of this element. Therefore, much more information concerning phytotoxicity and tolerance can be found for other widespread toxic trace metals (Cd, Pb, Cr, Cu) than for Ni [18,19]. White mustard, chosen as an object of this study, may tolerate excessive concentrations of trace metals, including Ni. This species is recognized as more sensitive to excessive amounts of Ni than to Cu or Zn [20,21].

White or yellow mustard (*Sinapis alba* L.) is believed to be native to the Mediterranean region, but nowadays is extensively cultivated throughout the world, with Canada and Nepal as the global leaders (around 52% of the world production in the 2015) as well as Ukraine, Russia, the Czech Republic, Italy, the UK, and the Netherlands as the European leaders [22]. White mustard seeds in food industry serve to produce table mustard, oil, and many kinds of spices. They are also used in the pharmaceutical and cosmetics industry. Furthermore, young fresh mustard raw leaves are used to made salad or juice [22,23]. In agriculture and horticulture, mustards are commonly used as green manure, fodder crop as well as winter or rotational cover crops in production of many species. Mustards may control weeds and a range of soil-borne pests, pathogens, and diseases. This is due to providing allelopathic compounds in the “biofumigation” process related to release of volatile toxic isothiocyanate compounds (ITCs) through the degradation of glucosinolates (GLS) [24,25].

Due to GLS synthesis in mustard, similar to the other members of the Brassicaceae family, the species is characterized by high S requirement, which are at least twice as high as that of cereal crops, especially at the flowering stage, since S is a constituent of sulfuric amino acids needed for the synthesis of seed proteins. This macronutrient is also an important constituent of lipids, polysaccharides, vitamins, and cofactors [26,27,28,29,30,31,32,33]. Besides building proteins and involvement in metabolism of secondary products, S is required for chlorophyll synthesis as well as proper cell metabolic pathways such as electron transport in Fe-S clusters, redox cycle, protein disulfide bonds. Sulphur deficiency in the environment and hence reduced yield and quality is a global problem related to progressive reduction of emissions of S compounds, common application of S-free NPK fertilizers, immobilization in soil and limited availability of S to plants, and much more intensive crop production [34,35]. An adequate S level is crucial not only for a proper plant growth and development, but also for enhanced resistance to various environmental stresses [36,37]. Plants have developed various strategies to cope with excess of trace metals. One of the strategies is induction of ligands synthesis, which are able to bind most trace metals in order to protect metal-target i.e., sensitive cellular organelles. Nickel is classified as a transition metal capable of binding to various types of naturally occurring phytocompounds. For instance, SH-containing ligands like reduced glutathione (GSH) or phytochelatins (PCs) form high-strength, durable complexes with trace metals. Nickel resistance is related mainly to GSH synthesis, since Ni is a very weak inductor of PCs synthesis. On the one hand, Ni is recognized as an important element for protecting plants against stressful conditions, among others, by participating in the regulation of the GSH pool involved in the defense against oxidative stress; on the other hand, Ni may induce oxidative stress [3,38,39,40].

Excessive Ni concentration disturbs the nutrient status in plants due to various unfavorable changes in their uptake and translocation [2,11,12,13,15], and there is evidence that S has a crucial role in enhancing tolerance to various types of stress [35]. Therefore, it may be possible to improve the nutrient status in Ni-stressed plants using S supplementation. This concerns especially species characterized by high S requirements, such as white mustard. In this study we investigated the impact of different S-SO_4_ concentrations on the micronutrient status in white mustard under short-term Ni exposition. We hypothesized that intensive S nutrition may improve the growth and micronutrient status of Ni-exposed white mustard by the modulation of selected physiological parameters of roots. It is obvious that only plant species that are able to survive in an environment containing excess of trace metals at their early (juvenile) growth stages may produce resistant and healthy adult individuals. These experiments are the part of a larger project concerning the possibility of enhancing plant resistance to stress induced by trace metals with the use of intensive S nutrition.

## 2. Results

### 2.1. Micronutrient Concentrations

The concentrations of micronutrient in the roots are presented in Table 1, while micronutrients in the shoots are presented in Table 2. The root and shoot B concentrations as well as the root Zn and shoot Cu level in the intensively S-supplied (6 or 9 mM) Ni-untreated plants were higher than those at the standard S level. In turn, the changes in the shoot Zn and root Cu concentrations, similar to the changes in the content of Fe, Cl, and Mo, were generally insignificant. The Mn concentration in plants supplied with the high S increased in the roots and decreased in the shoots, and these changes were more pronounced in the roots.

Increasing Ni concentrations in the nutrient solution, irrespective of the S level, generally resulted in a significant decline in Fe and B concentrations, which was much more pronounced in the shoots than in roots, and simultaneously did not change the Mo concentrations. Moreover, the statistical analysis of the main effects showed that the root Cu and Cl concentrations of Ni-exposed white mustard remained quite stable, whilst the shoot content of both these elements was substantially reduced. An exception was the decrease in the root Cu content recorded in plants treated with 0.04 mM Ni. Simultaneously, the root and shoot Zn concentration decreased and increased, respectively, whilst the concentration of Mn increased in both roots and shoots.

Intensive S nutrition, irrespective of the Ni concentration in the nutrient solution, generally caused increases in the shoot and root Fe, B, and Zn concentrations. However, the root and shoot Fe level in plants grown at 6 mM S and the root B concentration at 9 mM S did not change significantly. At the same time, root Cu and Mo concentrations remained quite stable, whilst their shoot concentrations were markedly elevated. In turn, the Mn concentration increased in roots and Cl level decreased in shoots.

The tendencies toward changes in the micronutrient concentrations for the interactions between the S and Ni (S × Ni) were generally similar to those above described for the main effects. However, a few differences were found. For instance, noteworthy is the decrease in the shoot Mn and Mo concentrations as well as the lack of significant changes in the root B and shoot Cl and Zn concentrations in the Ni-exposed plants grown at the standard S level. In turn, the following differences between the main effects and S × Ni interactions in the Ni-exposed plants supplied with extra S, in relation to plants treated with a comparable Ni concentration grown at standard S level, were found (see Table 1 and Table 2):

- no changes in the root Fe concentration under intensive S nutrition in plants under the lowest and the highest Ni exposure and the Fe increase under medium Ni concentration,
- no changes in the root B concentration under both elevated S levels,
- an increase in the shoot Mn at both the high S levels and a decrease in root Mn at the 9 mM S/0.0004 mM Ni treatment,
- an increase or no changes in the root Zn concentration at 6 and 9 mM S, respectively.

### 2.2. Micronutrient Translocation Factor (TF)

**Figure 1:**
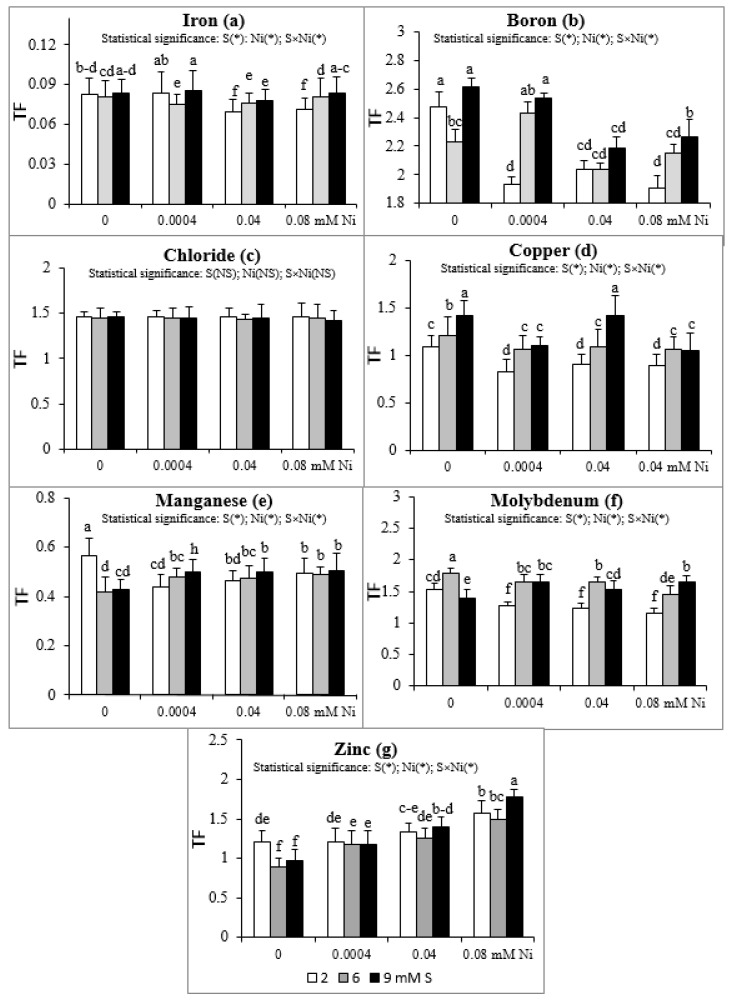
Translocation factor (TF) of micronutrients: (**a**) iron, (**b**) boron, (**c**) chloride, (**d**) copper, (**e**) manganese, (**f**) molybdenum, (**g**) zinc in white mustard “Rota” grown under different sulphur and/or nickel concentrations in the nutrient solution. Mean values (n = 9) followed by the same letter are not significant at 0.05 probability level based on Tukey’s honestly significance tests. Asterisks indicate significant effects for main factors and interactions between them.

Intensive S nutrition of plants non-exposed to Ni caused an increase of TF value for Cu (Figure 1d) and its reduction for Mn and Zn (Figure 1e,g). The Mo translocation from roots to shoots increased at 6 mM S and decreased at 9 mM S (Figure 1f). Simultaneously, the B translocation decreased at 6 mM S (Figure 1b). The presence of Ni in the nutrient solution at the standard S level severely reduced the TF value of Fe, B, Cu, Mn, and Mo without notable effect on Cl translocation (Figure 1a–f). The exception was no significant changes for the TF of Fe at the 2 mM S/0.0004 mM Ni treatment. Simultaneously, the TF value of Zn increased at the highest Ni concentration used, compared to the Ni-untreated plants (Figure 1g).

Supplementation with S of the Ni-exposed plants, in relation to the standard 2 mM S level, significantly elevated the TF of B, Cu, and Mo (Figure 1b,d,f) and did not notably affect the TF value for the Cl, Mn, and Zn (Figure 1c,e,g). Only the TF values of Mn and Zn at the 9 mM S/0.0004 mM Ni and 9 mM S/0.08 mM Ni treatments, respectively, were significantly higher than TF values found for the comparable Ni concentrations in the medium under the standard S level (Figure 1e,g). The lack of significant changes in TF value of B at the 6 mM S/0.04 mM Ni treatment was an exception (Figure 1b). In turn, the TF of Fe under the lowest Ni concentration used decreased at 6 mM S and did not change at 9 mM S, but increased under intensive S nutrition at the higher Ni concentrations (0.04 and 0.08 mM; Figure 1a).

### 2.3. Total Surface Charge (Q_tot_) and Cation Exchange Capacity (CEC)

**Figure 2:**
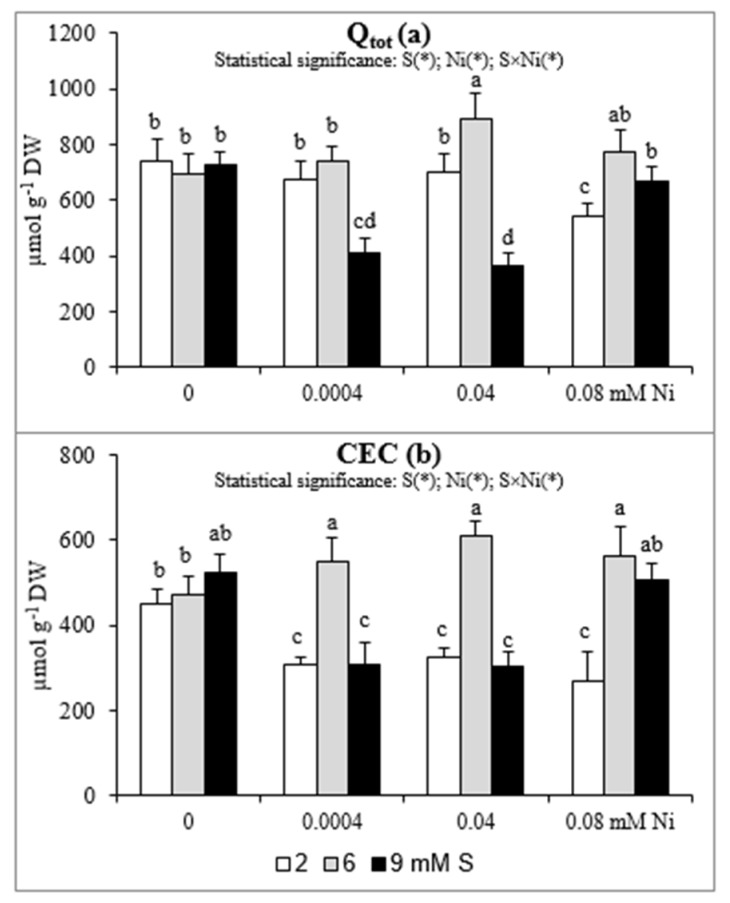
The (**a**) total surface charge (Q_tot_) and (**b**) cation exchange capacity (CEC) in white mustard “Rota” grown under different sulphur and/or nickel concentrations in the nutrient solution. Mean values (n = 9) followed by the same letter are not significant at 0.05 probability level based on the Tukey’s honestly significance tests. Asterisks indicate significant effects for main factors and interactions between them.

The changes in Q_tot_ and CEC are presented in Figure 2a,b. The CEC values at 2 mM S were markedly lower (by 29–40%) in roots of plants treated with Ni than in those non-exposed to this metal. Under these conditions the Q_tot_ decreased significantly (by 27%) only at the highest Ni concentration. When the Ni-exposed plants were supplied with extra S at the concentration of 6 mM the CEC increased by 80–89% in comparison to plants grown at standard S level. Meanwhile, the Q_tot_ value increased significantly for plants treated with 0.04 mM Ni supplied with 6 mM S and plants treated with 0.08 mM Ni and supplied with 6 or 9 mM S. In general, the impact of the highest concentration of S on CEC and Q_tot_ was negative at the lowest and moderate Ni concentrations used and their values were significantly reduced. On the other hand, under the highest Ni concentration the intensive S nutrition at 9 mM caused an increase in both CEC and Q_tot_ values. It is worth noting that the CEC in plants exposed to 0.08 mM Ni increased almost twice under intensive S nutrition in comparison to standard S level.

### 2.4. GSH and PCs Accumulation in Roots

**Figure 3:**
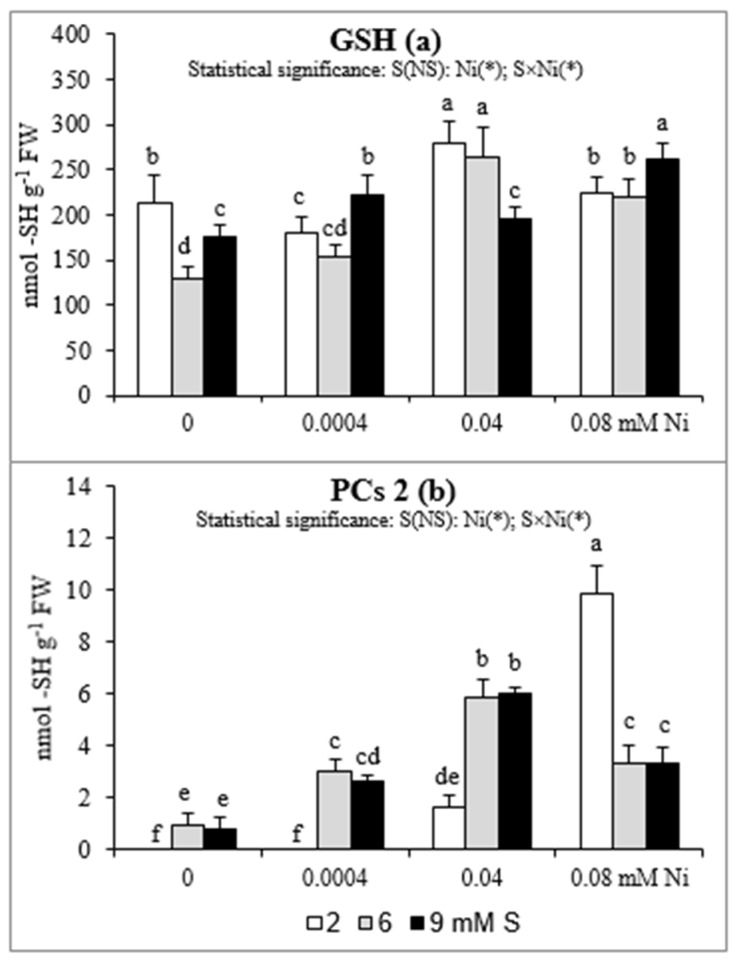
Concentrations of (**a**) reduced glutathione (GSH) and (**b**) phytochelatins (PCs) in white mustard “Rota” grown under different sulphur and/or nickel concentrations in the nutrient solution. Mean values (n = 9) followed by the same letter are not significant at 0.05 probability level based on Tukey’s honestly significance tests. Asterisks indicate significant effects for main factors and interactions between them.

The changes in root GSH concentrations were ambiguous (Figure 3a). In general, in Ni-exposed plants an elevated GSH accumulation was found, when we compare the level of this compound in the control plants and in these grown under presence of Ni at the appropriate S levels. The intensive S nutrition of Ni-untreated plants resulted in the decrease in GSH accumulation. At 6 mM S, no significant changes in GSH content were found at any of the tested Ni concentrations, in comparison to 2 mM S. In turn, the extra S supply at the concentration of 9 mM increased the GSH concentration in the plants treated with 0.0004 or 0.08 mM Ni, but decreased in those exposed to 0.04 mM Ni.

In roots of white mustard only small amounts of PCs 2 were found. Phytochelatins were not detectable or only trace concentrations of PCs 2 appeared in the roots of plants grown at 2 mM S without Ni or at the lowest Ni concentration. The enhanced concentrations of PCs 2 in the root tissues were detected with the increasing concentration of Ni. However, the intensified S nutrition significantly enhanced PCs 2 accumulation only at the lowest and the medium Ni concentration. Under the highest Ni exposition, the PCs 2 concentration decreased in comparison to the standard S level (Figure 3b).

### 2.5. Root and Shoot Biomass

**Figure 4:**
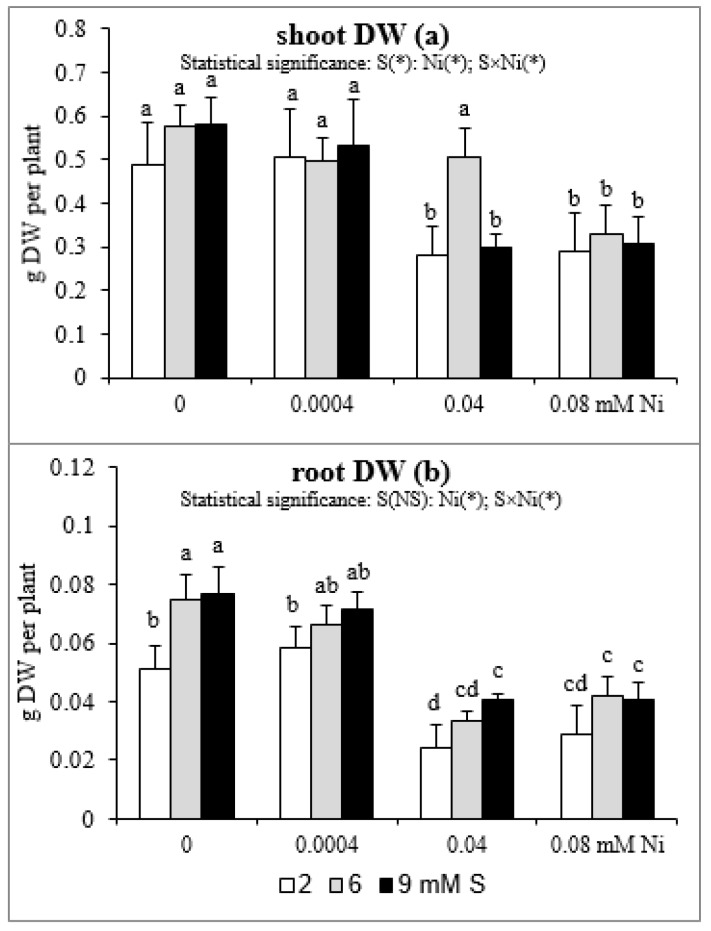
The dry weight (DW) of (**a**) shoots and (**b**) roots of white mustard “Rota” grown under different sulphur and/or nickel concentrations in the nutrient solution. Mean values (n = 36) followed by the same letter are not significant at 0.05 probability level based on Tukey’s honestly significance tests. Asterisks indicate significant effects for main factors and interactions between them.

The results of the influence of differentiated Ni and S concentrations in the nutrient solution on shoot and root DW are presented in Figure 4. Both the shoot and root biomass of plants treated with 0.04 or 0.08 mM Ni was reduced. Meanwhile, the lowest concentration of this element has no effect on plant growth. In mustard exposed to 0.04 mM Ni, the intensive S nutrition at 6 mM, in comparison to the standard S level, caused an increase in the shoot DW, which was not statistically different from Ni-untreated plants. However, this phenomenon did not occur at 9 mM S and under the highest Ni concentration used (Figure 4a). In turn, in mustard not subjected to Ni, the extra S supply induced a 45–50% increase in root DW compared to the standard S level. Such a trend was also observed in the presence of Ni, but it was not statistically confirmed. The exception was a significant increase in root DW of plants treated with 0.04 mM Ni under the influence of 9 mM S (Figure 4b).

## 3. Discussion

The lowest Ni concentration used (0.0004 mM) is claimed to be the highest value acceptable for the ground water and soil solution [6,41,42,43]. That means that the presence of higher concentrations of this metal, including those examined in our study (0.04 and 0.08 mM), requires implementation of conservation measures. The impact of Ni on the white mustard micronutrient status under the standard S dose (2 mM) are in agreement with the widely known statement that interference with other essential metal ions are an indirect pathway of Ni phytotoxicity. It was also confirmed that Ni-induced changes in the nutrient bioconcentrations are not only species-specific, but also unpredictable and contradictory. Additionally, these changes may be different in the roots and aboveground parts of plants [13,15,44]. The phenomenon of Ni-induced alterations in essential nutrient absorption, uptake, and transport and hence the disturbance in ionic homeostasis is a consequence of competition between Ni^2+^ and other cations (Cu^2+^, Fe^2+^, Mn^2+^, and Zn^2+^) for common binding sites as a result of similar characteristics, including comparable ionic radii [44,45,46]. Passive diffusion and active transport are recognized as two main mechanisms of Ni ions uptake by plants. Absorption of soluble Ni compounds occurs passively via a cation transport system. Chelated Ni compounds are taken up through secondary active-transport-mediated means, i.e., permeases. In turn, endocytosis is recognized as a mechanism through which insoluble Ni compounds primarily enter plant root cells. After absorption by roots, Ni transport to the shoot occurs very easily via the xylem. The processes of Ni transport and retranslocation are strongly regulated by metal-ligand complexes (nicotianamine, histidine, and organic acids) and by some specific Ni-binding proteins [6,13,19]. Moreover, Ni uptake and translocation occur with involvement of a Zn/Fe ZRT1/IRT1–ZIP transporter and a Mn ion transporter NRAMP [19,43,44]. The antagonism between Ni^2+^ ions and Cu^2+^ and Zn^2+^ ions suggests that these three elements are absorbed at the same site on the transporter [44]. Hence, the decreased Fe, Zn, and Cu concentrations in the mustard biomass, manifested as various types of chloroses, were also recorded by other researchers in many other plant species [41,45]. The results of the present study indicate antagonism between Ni and Cl and between Ni and Mo. It should be remembered that Ni may not only compete with essential nutrients, mainly Fe, Zn, and Cu, but also inhibit their functions. Nickel may replace the essential metal of metalloproteins and bind the residues of non-metalloenzymes. The binding of Ni ions outside the catalytic site of an enzyme induces allosteric modulation and, hence, the inhibition of the enzyme. Besides the above-mentioned indirect effect of Ni on enzyme activity, i.e., competitive inhibition of nutrient absorption and transport, a direct mechanism associated with strong affinity of Ni for the functional –SH groups of proteins is also known [6,13,19]. Moreover, the Ni-induced changes in the micronutrient status recorded in the present study may be explained by the disturbances in the cell membrane permeability caused by changes in the composition of sterols and phospholipids and changes in the structure and/or activity of cell membrane enzymes, mainly the H^+^-ATPase, which plays a key role in the active uptake and transport of essential nutrients [8,15,19].

The standard S concentration (2 mM) used in our experiments is recognized as a moderate level for this macronutrient. The S-SO_4_^2−^ concentration in the natural environment, i.e., unpolluted with trace metals, in arid regions and in soil solutions with residues of sulfide ore mine is in the range of 0.16–7, 3–16, and 13–110 mM, respectively [46]. Our study concerning the micronutrient bioconcentration under high S-SO_4_ levels (6 or 9 mM S) in mustard grown without Ni showed a synergistic effect between S and B, S and Cu, and S and Zn. Simultaneously, an antagonistic relationship between S and Cl was found. In turn, the S and Mn relationships were antagonistic at 6 mM S and synergistic at 9 mM S. It is claimed that an optimal S level increases the uptake of Mn and Zn [47]. The tendencies of changes in the root and shoot concentration of Mn, Cu, and Zn under intensive S nutrition in Ni-untreated white mustard were similar to those noted for this species in a field experiment of Jankowski et al. [48]. In their research the content of these three micronutrients in roots and shoots of Indian mustard was not affected by S fertilization, except for the Cu decrease in the shoots. However, in this study, the content of the other micronutrients in examined mustard species was not estimated. Moreover, in our previous study, an increase in the all macronutrient concentrations in roots and the S, K, and Ca level in shoots in Ni-exposed mustard supplied with extra S was found [49].

Taking into account the TF value, it may be concluded that white mustard has a strong ability to translocate B, Cl, Mo, and Zn from roots to shoots (TF > 1) and a weak ability to translocate Fe and Mn (TF < 1). This tendency was generally revealed irrespective of both the experimental factors, i.e., the S and Ni concentrations in the nutrient solution. Only the intensive S nutrition of Ni-untreated plants, compared to the standard S dose, strongly limited the translocation of Zn, reducing the Zn TF below 1. The Cu transfer within organs depended on the S and Ni concentrations in the nutrient solution. The Ni-exposed plants growing at 2 mM S showed a Cu TF value lower than 1 (about 0.9), in comparison with values exceeding 1 in plants grown without Ni. This implies that the Ni presence in the nutrient solution containing standard S levels reduced the Cu translocation. Simultaneously, the intensive S nutrition of Ni-treated mustard enhanced the ability to transfer Cu to shoots (Cu TF higher than 1). The micronutrient TF value obtained in our studies oscillated within the range of 0.070–0.086 (Fe), 1.91–2.611 (B), 1.42–1.46 (Cl), 0.82–1.41 (Cu), 0.42–0.57 (Mn), 1.16–1.80 (Mo), and 0.89–1.58 (Zn).

The electric charge is most frequently studied root surface property to describe the root CEC, its changes with soil pH, balance of plant cations of different valence and toxicity of trace metals. Electric charge of the root compartments is dominated by negatively charged groups, thus positively charged cations, including essential nutrients, accumulate near the roots surface [50]. In our study we have noticed a positive effect of intensive S nutrition of Ni-exposed plants on the studied properties of roots (Q_tot_ and CEC), especially at 6 mM S. The beneficial effect of extra S supply of Ni-treated mustard on the changes in the studied root properties may contribute to a better uptake of micronutrients and thus positively affect the mineral status of plants, which consequently stimulates their growth. On the other hand, in plants grown at 9 mM S, the values of Q_tot_ and CEC decreased at 0.004 and 0.04 mM Ni, but increased at 0.08 mM Ni in comparison to those non-treated with Ni. We suppose that under high Ni bioconcentration, the higher S levels can be required to abolish toxic effect of Ni to the studied root parameters.

The antioxidant defense is believed to play a key role in the Ni tolerance, as the oxidative stress induction and the disturbances in the nutrient status are the major mechanism of the phytotoxicity of this element [13]. Also, there is no doubt that Ni-tolerance is linked to S metabolism, primarily with high levels of O-acetyl-L-serine (OAS), Cys, and GSH associated with the high activity of Ser acetyl transferase (SAT). Nickel is recognized as an element with strong ability to bind to various types of chelating agents, especially S-donor ligands rich in highly reactive S functional groups. However, Ni is recognized as a weak PCs inductor [4,6,51,52,53], which has been also confirmed in our study. Although the amount of PCs in plants exposed to Ni was not high, but in the presence of this metal the level PCs 2 significantly increased and the intensive S nutrition enhanced PCs 2 accumulation under the lowest and moderate Ni exposure. On the other hand, there are strong evidence that Ni may play a role in plant response to stressful conditions by decreasing the level of methylglyoxal (MG; a toxic, mutagenic alpha-ketoaldehyde) as well as participating in the regulation of the GSH homeostasis. In the degradation pathway of MG are involved glyoxalases I and II (GLY-I and II) and recently it was found that Ni ions may activate GLY-I in plants. The GLY-I dependence on Ni may play an additional role in the regeneration of GSH [3,54]. In our study the concentrations of GSH at the standard S level increased under moderate Ni exposure but not under both the lowest and the highest concentrations of this metal. We suppose that the concentration of 0.0004 mM Ni could be too low to effectively induce GSH accumulation but the concentration 0.08 mM Ni could have an adverse effect on GLY-I activity and therefore no increase in GSH concentration was found. The intensive S nutrition at 9 mM S caused enhanced accumulation of GSH only under the lowest and the highest Ni exposure. This effect was not observed at 6 mM S. Therefore, the beneficial effect of intensive S fertilization of Ni-stressed white mustard, which manifested itself as a rise in the Fe, Mn, and Zn bioconcentration, especially in the shoot biomass, is probably related to positive changes in root surface property as CEC, but not with an increase in GSH or PC synthesis in root tissues.

## 4. Materials and Methods

### 4.1. Plant Material and Growth Conditions

The biological object of the study was white mustard (*Sinapis alba* L.) “Rota” (Brassicaceae). Seven-day-old homogenous, healthy seedlings obtained from seeds germinated on quartz sand moistened with distilled water were transferred to 1 L glass jars (two plants per each jar) filled with full-strength Hoagland’s solution No. 2 with different levels of S and Ni. A combination of three S levels (standard: 2 mM S, and intensive: 6 or 9 mM S; sulfate VI (S-SO_4_)) and four Ni concentrations (0, 0.0004, 0.04, and 0.08 mM Ni; NiCl_2_) was used to arrange the experimental treatments. In all experimental treatments, the standard S dose (2 mM) was supplied as MgSO_4_ and supplemented with corresponding amounts of Na_2_SO_4_. The dose of S applied as Na_2_SO_4_ for the level of 2, 6, and 9 mM were 0, 4, and 7 mM, respectively. In each treatment, the levels of Na and Cl were equalized and the pH of the nutrient solution was set at 5.8–6.0 by adding appropriate amounts of diluted solutions of NaCl or HCl. The plants were cultured in a controlled-climate vegetation room at 14-h day length, PPF of 250–270 μmol × m^−2^ × s^−1^ at the level of the tops of the plants, temperature 25/20 °C (day/night), and relative air humidity of 50–60%. The nutrient solution was aerated for 15 min. every three days and replenished with a fresh nutrient solution when the medium level was depleted to ca. 70% of the initial level. After 14 days of vegetation under the differentiated S and Ni concentrations, the plants were harvested and subjected to the analysis.

### 4.2. Determination of Biomass and Micronutrient Concentrations

The roots and shoots of twelve randomly selected plants were dried in a forced air oven at 105 °C for 48 h, their dry weight (DW) were determined, and the samples were subjected to the analysis of the micronutrient concentration. The dry plant samples were ground to form a powder using a laboratory grinding mill. The total content of Fe, Mn, Zn, Mo, and Cu in roots and shoots were analyzed by atomic-absorption spectrophotometry (AAS) after wet mineralization with sulfuric acid (VI) and perhydrol [55,56]. To measure the B concentration, the Azomethine-H method was employed and the absorbance was read by spectrophotometry at 410 nm [57]. The Cl concentration was determined by the nephelometric method using nitric acid and silver nitrate [58]. The data obtained were used to calculate the value of the translocation factor (TF) of micronutrients (defined as a quotient of concentration of a given element in shoots and its concentration in the roots).

### 4.3. Determination of CEC and Q_tot_ by Potentiometric Titration

The values of CEC under differentiated experimental conditions were determined using potentiometric titration described in detail by Szatanik-Kloc et al. [50]. In brief, the fresh roots were placed in a ventilated room at 30 °C for 48 h. Then, a suspension of plant roots equilibrated overnight with 1 M L^−1^ NaCl was adjusted to pH = 3.0 until the pH was stable over the next 5 min and titrated automatically (Titrino 702 MS, Metrohm AG, Switzerland) by 60 s increments of 1 μL 0.100 M L^−1^ sodium hydroxide solution to pH = 10. The surface charge at pH = 7 was taken as the root CEC and the charge at pH = 10 was considered as the total surface charge (Q_tot_).

### 4.4. Determination of γ-Glu-Cys Peptides by HPLC Method

The determine the GSH and PCs concentrations the root samples were weighted and ground in an ice-cooled mortar with a double volume of 0.1 M HCl. The crude assay solution was obtained by homogenate centrifugation at 14 000 rpm at 4 °C (3 times by 5 min). Beckman chromatograph (model 126/166) with Supelco precolumn (4.6 × 10 mm) and column (4.6 × 250 mm) (both filled with Ultrasphere C18) were used. The peptide solution (100 µL) was separated in a linear gradient (0–20%) of acetonitrile (ACN) in 0.05% trifluoroacetic acid (TFA) and was subjected to a post-column reaction with 200 µM 5,5′-dithiobis-2-nitrobenzoic acid (DTNB) in the 0.05 M potassium-phosphate buffer (pH = 7.6). The absorbance of the resulted reaction products was measured at 405 nm using a Beckman detector (model 166). The chromatograms were analysed using Karat 7.0 software (Beckman).

### 4.5. Statistical Analysis

The results were processed statistically using analysis of variance (ANOVA) for a two-factor experiment (3 levels of S and 4 concentrations of Ni in the nutrient solution) established in a completely randomized design, using Statistica 9.0 software. Each of the twelve experimental treatments included twenty replications (20 jars with 2 plants in each) and the whole experiment was repeated independently three times under the same conditions. This means that each of the twelve experimental treatments included in total 60 jars and 120 plants. The main effects of the S level and Ni concentration were compared using Tukey’s multiple comparison test at the significance level *p* ≤ 0.05. The comparison of the values within the same treatment as well as the mean values in each of the twelve treatments collected from each of the three independent replicates of the experiment over the time did not show statistically proven differences.

## 5. Conclusions

The results from the present study show that it is possible to prevent to some extent unfavorable changes in the micronutrient status of the white mustard “Rota” exposed to Ni (0.0004–0.08 mM) with the use of intensive S-SO_4_ nutrition (6 or 9 mM S). Generally, an increase in the Fe, Mn, and Zn bioconcentrations, especially in the shoots, were found in Ni-treated plants supplied with extra S. The elevated concentrations of shoot B, Cu, and Mn were also revealed, without changes in their root concentrations. Simultaneously, the shoot Cl concentrations decreased. Furthermore, intensive S nutrition of Ni-exposed mustard, in relation to the standard 2 mM S dose, in general, enhanced the translocation of Fe, Cu, Mo, and B from roots to shoots. The improved micronutrient status of Ni-treated mustard supplied with extra S can be related to positive changes in total surface charge and cation exchange capacity of roots. However, an increase in the shoot biomass was noted only at 6 mM S in plants exposed to 0.04 mM Ni. These results contribute to the knowledge concerning mechanisms employed by plants intensively supplied with S to cope with Ni stress. The present studies offer an opportunity to increase the resistance of white mustard to Ni excess using an intensified S nutrition. These methods are quite promising and effective, easy to apply, as well as sustainable and safe for the environment. It may find practical application, which is especially important for farmers and horticulturalists, but needs further confirmation under field conditions.

## Figures and Tables

**Table 1:** The concentration of micronutrients in the root biomass of white mustard grown for two weeks under different sulphur and/or nickel concentrations in the nutrient solution.

Concentration in the Nutrient Solution (mM)		Concentration of the Micronutrients in the Roots (mg kg^−1^ DM)						
S	Ni	Fe	B	Cl	Cu	Mn	Mo	Zn
2		1378 ± 42.6^a^	17.24 ± 1.58^c^	8485 ± 53.87	5.48 ± 0.22^ab^	47.18 ± 1.02^e^	0.642 ± 0.093	34.89 ± 0.95^d–f^
6	0.00	1382 ± 43.0^a^	22.13 ± 2.73^a^	8478 ± 70.42	5.72 ± 0.35^a^	53.37 ± 0.97^bc^	0.585 ± 0.060	45.57 ± 1.14^a^
9		1377 ± 52.2^a^	20.77 ± 1.65^b^	8498 ± 55.39	5.13 ± 0.56^a–c^	55.74 ± 0.76^a^	0.663 ± 0.074	42.61 ± 0.99^ab^
2		1372 ± 32.5^a^	18.14 ± 2.51^c^	8492 ± 97.01	5.01 ± 0.46^a–d^	54.81 ± 0.84^a–c^	0.608 ± 0.087	36.28 ± 1.22^de^
6	0.0004	1380 ± 61.7^a^	17.29 ± 1.67^c^	8484 ± 41.66	5.36 ± 0.70^ab^	53.78 ± 0.66^a–c^	0.597 ± 0.058	40.67 ± 1.48^bc^
9		1370 ± 62.7^a^	17.71 ± 2.49^c^	8503 ± 84.72	5.29 ± 0.39^a–c^	51.13 ± 0.80^d^	0.591 ± 0.051	38.52 ± 1.09^b–d^
2		1263 ± 21.9^c^	17.05 ± 2.63^c^	8473 ± 72.38	4.42 ± 0.31^cd^	53.19 ± 0.75^c^	0.617 ± 0.052	31.12 ± 1.14^h^
6	0.04	1279 ± 42.0^c^	18.09 ± 2.57^c^	8488 ± 89.84	4.73 ± 0.66^b–d^	55.31 ± 0.88^ab^	0.580 ± 0.041	35.43 ± 0.99^de^
9		1310 ± 30.4^b^	17.42 ± 1.66^c^	8482 ± 75.05	4.16 ± 0.54^d^	54.02 ± 0.76^ac^	0.635 ± 0.049	33.74 ± 1.16^e–h^
2		1307 ± 36.1^b^	18.59 ± 1.67^c^	8500 ± 59.32	4.87 ± 0.57^a–d^	51.04 ± 0.64^d^	0.659 ± 0.102	28.13 ± 0.94^hi^
6	0.08	1299 ± 33.2^b^	17.35 ± 1.48^c^	8477 ± 93.41	5.07 ± 0.61^a–c^	54.28 ± 0.93^a–c^	0.668 ± 0.044	31.37 ± 1.21^f–h^
9		1313 ± 42.2^b^	18.28 ± 2.53^c^	8492 ± 84.85	5.34 ± 0.43^ab^	52.95 ± 0.75^cd^	0.584 ± 0.057	26.48 ± 0.87^i^
***Main effects***								
*S*								
*2*		*1330 ± 52.4*	*17.76 ± 1.48^b^*	*8488 ± 70.21*	*4.95 ± 0.37*	*51.56 ± 0.43^b^*	*0.632 ± 0.027*	*32.61 ± 0.73^c^*
*6*		*1335 ± 41.4*	*18.72 ± 1.52^a^*	*8482 ± 82.08*	*5.22 ± 0.23*	*54.19 ± 0.41^a^*	*0.608 ± 0.024*	*38.26 ± 1.64^a^*
*9*		*1342 ± 59.0*	*18.55 ± 1.41^ab^*	*8494 ± 70.83*	*4.98 ± 0.69*	*53.46 ± 0.48^a^*	*0.618 ± 0.019*	*35.34 ± 0.68^b^*
*Ni*								
*0*		*1379 ± 44.8^a^*	*20.05 ± 1.64^a^*	*8487 ± 91.37*	*5.44 ± 0.45^a^*	*52.10 ± 0.67^c^*	*0.630 ± 0.037*	*41.02 ± 0.70^a^*
*0.0004*		*1374 ± 51.3^a^*	*17.71 ± 1.58^b^*	*8493 ± 72.44*	*5.22 ± 0.68^a^*	*53.24 ± 0.48^ab^*	*0.599 ± 0.018*	*38.49 ± 0.75^b^*
*0.04*		*1283 ± 32.4^b^*	*17.52 ± 1.46^b^*	*8481 ± 63.36*	*4.44 ± 0.47^b^*	*54.17 ± 0.41^a^*	*0.611 ± 0.022*	*33.43 ± 0.63^c^*
*0.08*		*1306 ± 42.3^ab^*	*18.07 ± 1.53^b^*	*8490 ± 70.09*	*5.09 ± 0.64^a^*	*52.76 ± 0.45^bc^*	*0.637 ± 0.028*	*28.66 ± 0.59^d^*
*Statistical significance*								
*S*		*NS*	*	*NS*	*NS*	*	*NS*	*
*Ni*		*	*	*NS*	*	*	*NS*	*
*S × Ni*		*	*	*NS*	*	*	*NS*	*

Note: The results are presented as the mean ± SD of nine measurements (three measurements made per each of three independent repetition of the experiment over time). Means (n = 9) sharing the same letter in a column do not differ significantly according to Tukey’s multiple range test at *p* ≤ 0.05; significant effects for the main factors and for interaction between them are indicated with asterisks (*); NS—non-significant.

**Table 2:** The concentration of micronutrients in the shoot biomass of white mustard grown for two weeks under different sulphur and/or nickel concentrations in the nutrient solution.

Concentration in the Nutrient Solution (mM)		Concentration of the Micronutrients in the Shoots (mg kg^−1^ DM)						
S	Ni	Fe	B	Cl	Cu	Mn	Mo	Zn
2		114.27 ± 3.29^ab^	42.73 ± 2.89^cd^	12,359 ± 85.2^a^	5.96 ± 0.19^b^	26.72 ± 1.55^a^	0.983 ± 0.057^a^	42.15 ±0.87^c–e^
6	0.00	111.86 ± 2.91^ab^	49.36 ± 1.14^b^	12,311 ± 98.5^bc^	6.88 ± 0.15^a^	22.45 ± 1.63^e^	1.052 ± 0.071^a^	40.73 ± 1.11^e^
9		115.09 ± 3.84^ab^	54.22 ± 2.93^a^	12,348 ± 60.3^ab^	7.25 ± 0.26^a^	23.96 ± 1.72^de^	0.928 ± 0.049^a–d^	41.06 ± 1.03^e^
2		115.24 ± 4.09^a^	35.07 ± 1.28^g^	12,351 ± 97.1^ab^	4.12 ± 0.34^d^	24.02 ± 1.53^d^	0.770 ± 0.078^b–d^	43.87 ± 0.75^b–d^
6	0.0004	103.26 ± 3.71^c–e^	42.06 ± 1.05^d^	12,301 ± 63.0^c^	5.68 ± 0.26^b^	25.69 ± 1.47^a–c^	0.981 ± 0.043^a^	47.67 ± 1.17^a^
9		117.74 ± 3.35^a^	44.85 ± 1.35^c^	12,275 ± 60.3^c^	5.81 ± 0.21^b^	25.53 ± 1.61^a–c^	0.975 ± 0.065^a^	45.38 ± 1.21^ab^
2		88.22 ± 5.29^g^	34.72 ± 1.07^g^	12,385 ± 51.4^a^	4.01 ± 0.28^d^	24.72 ± 1.55^cd^	0.759 ± 0.057^d^	41.75 ± 1.09^de^
6	0.04	97.29 ± 4.07^ef^	36.94 ± 1.22^fg^	12,149 ± 88.7^d^	5.16 ± 0.20^bc^	26.30 ± 1.59^ab^	0.960 ± 0.048^a–c^	44.29 ± 0.86^bc^
9		101.56 ± 5.61^de^	38.12 ± 1.24^f^	12,298 ± 36.0^c^	5.92 ± 0.24^b^	26.91 ± 1.47^a^	0.972 ± 0.061^a^	47.17 ± 1.14^a^
2		93.81 ± 4.48^fg^	35.48 ± 1.79^fg^	12,377 ± 51.6^a^	4.35 ± 0.21^cd^	25.19 ± 1.62^b–d^	0.763 ± 0.066^cd^	44.31 ± 0.92^bc^
6	0.08	104.77 ± 3.02^cd^	37.35 ± 2.14^fg^	12,304 ± 68.3^c^	5.37 ± 0.22^b^	26.73 ± 1.53^a^	0.970 ± 0.072^ab^	46.97 ± 1.17^a^
9		110.01 ± 5.23^bc^	41.37 ± 1.33^d^	12,092 ± 54.2^e^	5.59 ± 0.31^b^	26.70 ± 1.58^a^	0.967 ± 0.054^ab^	47.06 ± 1.20^a^
***Main effects***								
*S*								
*2*		*102.89 ± 4.61^b^*	*37.00 ± 1.98^c^*	*12,368 ± 78.2^a^*	*4.61 ± 0.14^b^*	*25.16 ± 1.44*	*0.819 ± 0.037^b^*	*43.02 ± 0.47^b^*
*6*		*104.30 ± 3.57^b^*	*41.43 ± 1.45^b^*	*12,266 ± 87.1^ab^*	*5.77 ± 0.11^a^*	*25.29 ± 1.52*	*0.991 ± 0.029^a^*	*44.92 ± 0.44^a^*
*9*		*111.10 ± 4.79^a^*	*44.64 ± 1.83^a^*	*12,253 ± 56.2^b^*	*6.14 ± 0.19^a^*	*25.78 ± 1.47*	*0.961 ± 0.031^a^*	*45.17 ± 0.50^a^*
*Ni*								
*0*		*113.74 ± 2.97^a^*	*48.77 ± 2.65^a^*	*12,399 ± 84.3^a^*	*6.70 ± 0.11^a^*	*24.38 ± 1.42^b^*	*0.988 ± 0.027*	*41.31 ± 0.78^c^*
*0.0004*		*112.08 ± 3.65^a^*	*40.66 ± 1.23^b^*	*12,309 ± 74.4^a^*	*5.20 ± 0.17^b^*	*25.08 ± 1.43^ab^*	*0.909 ± 0.022*	*45.64 ± 0.52^b^*
*0.04*		*95.69 ± 4.72^b^*	*36.59 ± 1.12^d^*	*12,277 ± 71.1^b^*	*5.03 ± 0.22^b^*	*25.98 ± 1.51^a^*	*0.897 ± 0.032*	*44.40 ± 0.47^b^*
*0.08*		*102.86 ± 4.53^a^*	*38.07 ± 1.76^c^*	*12258 ± 59.2^b^*	*5.10 ± 0.13^b^*	*26.21 ± 1.48^a^*	*0.900 ± 0.029*	*56.11 ± 0.51^a^*
*Statistical significance*								
*S*		*	*	*	*	*NS*	*	*
*Ni*		*	*	*	*	*	*NS*	*
*S × Ni*		*	*	*	*	*	*	*

Note: For explanation see Table 1.
